# Refusal of Adjuvant Therapies and Its Impact on Local Control and Survival in Patients with Bone and Soft Tissue Sarcomas of the Extremities and Trunk

**DOI:** 10.3390/cancers16020239

**Published:** 2024-01-05

**Authors:** Franziska Mentrup, Alexander Klein, Lars Hartwin Lindner, Silke Nachbichler, Boris Michael Holzapfel, Markus Albertsmeier, Thomas Knösel, Hans Roland Dürr

**Affiliations:** 1 Department of Orthopaedics and Trauma Surgery, Orthopaedic Oncology, Musculoskeletal University Center Munich (MUM), LMU University Hospital, LMU Munich, 81377 München, Germany; fmentrupfm@googlemail.com (F.M.); alexander.klein@med.uni-muenchen.de (A.K.); boris.holzapfel@med.uni-muenchen.de (B.M.H.); 2 SarKUM, Center of Bone and Soft Tissue Tumors, LMU University Hospital, LMU Munich, 81377 München, Germany; lars.lindner@med.uni-muenchen.de (L.H.L.); silke.nachbichler@med.uni-muenchen.de (S.N.); markus.albertsmeier@med.uni-muenchen.de (M.A.); thomas.knoesel@med.uni-muenchen.de (T.K.); 3 Department of Medicine III, LMU University Hospital, LMU Munich, 81377 München, Germany; 4 Department of Radiation Oncology, LMU University Hospital, LMU Munich, 81377 München, Germany; 5 Department of General, Visceral and Transplantation Surgery, LMU University Hospital, LMU Munich, 81377 München, Germany; 6 Institute of Pathology, LMU Munich, 81377 München, Germany

**Keywords:** sarcoma, surgery, radiotherapy, chemotherapy, decision-making, treatment refusal

## Abstract

**Simple Summary:**

In a cohort of 828 patients undergoing surgery for sarcomas of the extremities, pelvis, and trunk, approximately half of them were advised to receive radiotherapy. Notably, 4.7% declined the recommended treatment regimen, resulting in a decrease of both overall survival and local recurrence-free survival. Similarly, 40% of patients within the same group were advised to undergo chemotherapy; 8.8% of these patients rejected the recommendation, leading to a reduced local recurrence-free survival. Enhancing physician-patient communication and addressing underutilization of recommended therapies in sarcoma patients should be a priority for surgeons and oncologists alike.

**Abstract:**

Background: In soft tissue or bone sarcomas, multimodal therapeutic concepts represent the standard of care. Some patients reject the therapeutic recommendations due to several reasons. The aim of this study was to assess the impact of that rejection on both prognosis and local recurrence. Methods: Between 2012 and 2019, a total of 828 sarcoma patients were surgically treated. Chemotherapy was scheduled as a neoadjuvant, and adjuvant multi-agent therapy was performed following recommendations from an interdisciplinary tumor board. Radiotherapy, if deemed appropriate, was administered either in a neoadjuvant or an adjuvant manner. The recommended type of therapy, patient compliance, and the reasons for refusal were documented. Follow-ups included local recurrences, diagnosis of metastatic disease, and patient mortality. Results: Radiotherapy was recommended in 407 (49%) patients. A total of 40 (10%) individuals did not receive radiation. A reduction in overall survival and local recurrence-free survival was evident in those patients who declined radiotherapy. Chemotherapy was advised for 334 (40%) patients, 250 (75%) of whom did receive all recommended cycles. A total of 25 (7%) individuals did receive a partial course while 59 (18%) did not receive any recommended chemotherapy. Overall survival and local recurrence-free survival were reduced in patients refusing chemotherapy. Overall survival was worst for the group of patients who received no chemotherapy due to medical reasons. Refusing chemotherapy for non-medical reasons was seen in 8.8% of patients, and refusal of radiotherapy for non-medical reasons was seen in 4.7% of patients. Conclusions: Divergence from the advised treatment modalities significantly impacted overall survival and local recurrence-free survival across both treatment modalities. There is an imperative need for enhanced physician-patient communication. Reducing treatment times, as achieved with hypofractionated radiotherapy and with therapy in a high-volume sarcoma center, might also have a positive effect on complying with the treatment recommendations.

## 1. Introduction

In the treatment of soft tissue or bone sarcomas of the extremities, limb-sparing surgery has evolved as the standard, rendering amputation rarely necessary [1,2,3]. This paradigm shift has predominantly emanated from the integration of multimodal therapy concepts, including the use of chemotherapy (CTx), radiotherapy (RTx), and surgical interventions [2]. There are diverse methodological approaches available to orchestrate these modalities in various sequences, each with different advantages and disadvantages [4]. Neoadjuvant chemotherapy has not demonstrated negative effects on wound healing in sarcoma surgery unless surgery is performed during chemotherapy [5]. Hence, neoadjuvant and adjuvant chemotherapy protocols are widely recommended [6]. Radiotherapy has for decades been part of the multimodal concept [7], but in a neoadjuvant setting, wound healing disorders have to be expected [4].

However, many protocols in RTx or CTx necessitate a considerable time duration, compelling patients to endure the presence of the tumor in a neoadjuvant setting or to endure uncomfortable side effects. Both circumstances lead to a suboptimal utilization of the recommended effective sarcoma therapies. This has been mainly scrutinized through studies reliant on cancer registries such as the Surveillance, Epidemiology, and End Results (SEER) database, encompassing multiple types of cancers [8].

Recommendations for the use of multimodality therapy in sarcoma patients are increasing. In 2022, Nigam et al. showed that neoadjuvant therapy had increased by 7% per year, whereas surgery alone had decreased by 4% every year [9]. Factors predictive of surgery alone were older age, nonprivate insurance, increasing travel distance, and multimorbidity. Conversely, factors associated with neoadjuvant therapy and surgery were private insurance, higher education, care at academic or high-volume institutions, the size of the tumor being <5 cm, and having higher-grade tumors.

The principle objective of this study was to evaluate the outcome of the patients’ decisions for or against a recommended therapy on prognosis and local recurrence-free survival within a single-center cohort of sarcoma patients. Potential factors influencing these outcomes were described as recorded in the patients’ files, or if not collected at all, were evaluated by other studies mentioned in the discussion section.

## 2. Patients and Methods

This study included 828 patients surgically treated at our institution for a bone or soft tissue sarcoma with a curative therapeutic approach between 2012 and 2019. The mean age of the 448 (54%) male and 380 female (46%) patients was 55 years (2–99 years). A total of 51 patients were below 18 years of age. None of the study patients refused the recommended therapies. Pathology was performed for most of the cases in our center. If performed outside and regarded as inconclusive in the tumor board discussion, we had a second review performed at our center. In some studies, as for Ewing and osteosarcomas, a second review was mandatory and was performed by the reference pathologists of those studies. In selected cases, we sent the material for a further opinion to a second reference center. The distribution of histiotypes is summarized in Table 1, and all further patient details are listed in Table 2. A subset of these patients was included in a previously published study focusing on resection margins [9].

### 2.1. Surgery

All surgeries were performed by two experienced surgeons. In general, surgery aimed to attain an R0-resection, employing local or free flaps as warranted. In the case of bone tumor resections, different methods of reconstruction were applied.

R0 resection was achieved in 666 (80%) patients, whereas in 156 (19%) patients histopathological analyses confirmed an R1-resection. This was intended for the majority of patients, especially in cases of atypical lipomas (liposarcoma G1 of the extremities). Only 6 (1%) patients had an R2 resection.

### 2.2. Radiotherapy

Radiotherapy was administered either in a neoadjuvant or an adjuvant fashion. The decision regarding its application and its appropriate timing underwent careful consideration through individualized and interdisciplinary discussions as part of routine tumor board meetings. In general, in patients with high-grade soft tissue sarcomas in a deep location and a resection margin below 1 cm, as nearly always seen in some region near vessels, nerves, or the bone, radiotherapy was advised. Radiotherapy was also advised in all patients with myxoid liposarcoma. In superficial tumors, the decision was individualized, taking into account the size and location of the tumor as also the grading and resection margin. In bone tumors, additional radiotherapy was included for many patients with Ewing sarcomas as advised by the EURO-EWING [11] protocol. In osteosarcoma, radiotherapy was only taken into account in a R1-situation without possibility of further surgery.

### 2.3. Chemotherapy

In general, chemotherapy was scheduled as a combined neoadjuvant- and adjuvant-multi-agent therapeutic approach. For soft tissue sarcomas, this predominantly encompassed AI (Adriamycin and Ifosfamide) or EIA (Etoposid, Ifosamid and Adriamycin), and occasionally included other regimens, incorporating local hyperthermia for the majority of these cases [6,11]. In bone sarcomas, diverse protocols such as EURAMOS [12] and EURO-B.O.S.S. [13] were applied for osteosarcomas and bone sarcoma patients aged over 40, and EURO-EWING [14] was applied for Ewing sarcomas. Decisions regarding the administration and timing of chemotherapy were carefully tailored and discussed on a regular basis within an interdisciplinary tumor board. In general, all patients included with indications in those studies were given a recommendation for systemic therapy if no medical reasons such as cardiac or renal exclusion criteria, especially in aged persons, were evident. Due to the toxicity of the EURO-B.O.S.S. protocol in aged patients, individual decisions were made.

### 2.4. Psycho-Oncologic Advice

Based on needs, more than 10 psycho-oncologists were available to the center. They focused on support and treatment during and after sarcoma therapy, on conversations with relatives, and on giving advice on illness-related problems for the partner, family, and community. The team offered couple and family therapy as well as end-of-life and grief support. They provided further support services such as self-help groups, nutritional advice, social advice, and sports offers.

### 2.5. Endpoint and Statistics

For all patients, the recommended type of adjuvant therapy, acceptance or refusal, and the cause for refusal were documented. During follow-up, the evaluation encompassed local recurrences, diagnosis of metastatic disease, and the patient’s mortality.

Categorical variables were presented as absolute and relative frequencies, while continuous variables were presented as mean and standard deviation (SD). For comparative analyses between groups, two-tailed Student’s *t*-tests or Mann–Whitney U tests were used. The significance of differences in proportions was determined using the Chi-square or Fisher’s exact test. Tests were two-tailed with *p*-values of 0.05 or less considered to be statistically significant. Multiple logistic regression was performed to account for various confounding factors. The data analysis software employed for these analyses was MedCalc^®^ (Version 19.0.3).

## 3. Results

**Figure 1:**
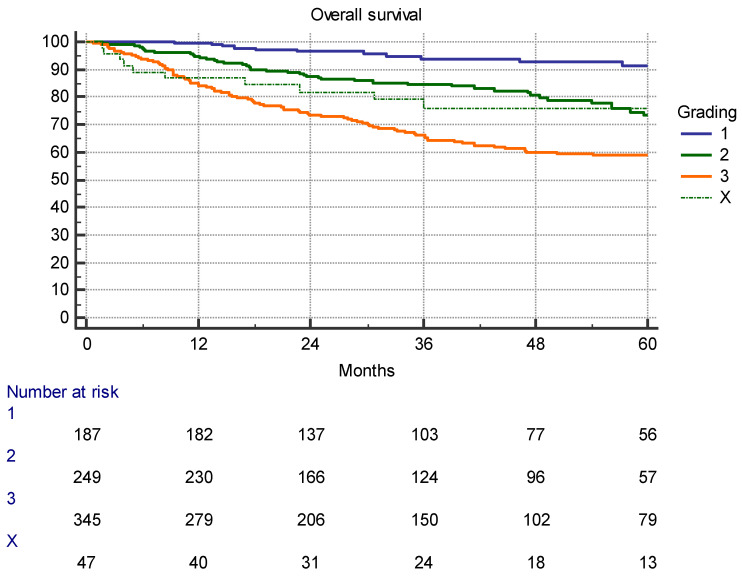
Overall survival in 828 patients after resection of the tumor (*p* < 0.0001).

A total of 632 of 828 (76.3%) analyzed patients were alive at the cut-off date. Median follow-up of those 632 survivors was 40.7 months (0.4–106.4 months). Only a minority of the 24 (3.8%) survivors were subject to a follow-up of less than 12 months. Overall, 196 patients died. Cause of death was not documented in our files. Therefore, disease-specific survival could not be calculated. For a gross overview of outcomes, overall survival in relation to tumor grading is shown in Figure 1.

### 3.1. Radiotherapy

Neoadjuvant radiotherapy was recommended in 121 patients (15%). Only 5 (4%) of those to whom it was recommended refused or did not undergo the recommended regimen. In three patients, this was caused by tumor progression, and in one case each, local wound impairment and rejection of therapy were mentioned.

Adjuvant radiotherapy was recommended in 286 (35%) cases. A total of 252 (88% of those to whom it was recommended) willingly received the indicated treatment. Conversely, 18 patients (6%) refused radiotherapy. Other reasons for abstaining from radiotherapy included death of the patient (n = 7), wound complications (n = 7), progression of disease (n = 1), or reasons that were unknown (n = 2).

In total, radiotherapy was advised for 407 (49%) patients, 40 (10%) of whom did not undergo radiation due to various reasons. A total of 181 from the 311 (58.2%) patients below 65 years had a recommendation for RTx, while only 226 of the 517 (43.7%) patients >65 years had the same (*p* = 0.0001). The mean age of patients who received the recommended radiotherapy was 58 years, whereas in those who declined RTx, it was 67 years (*p* < 0.006). There was no difference between patients with bone and soft tissue sarcomas.

**Figure 2:**
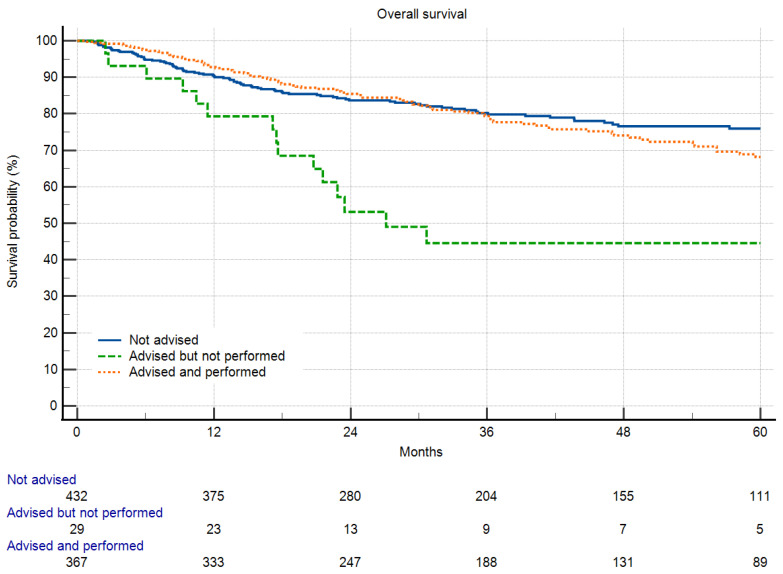
Overall survival in respect to radiotherapy and refusal of radiotherapy (*p* < 0.0001). Patients with RTx not advised and those with RTx advised and performed showed no significant difference. Patients with advised and performed RTx did better than those who declined RTx (*p* < 0.0001).

**Figure 3:**
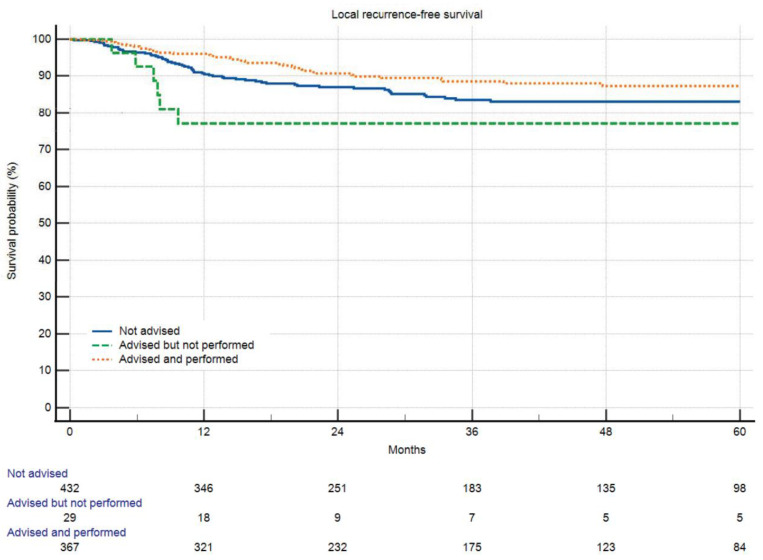
Local recurrence-free survival in respect to radiotherapy and refusal of radiotherapy (*p* < 0.0001). There was no significant difference between patients with advised and performed RTx and those without advised RTx. Patients who refused radiotherapy did worse than those with RTx advised and performed (*p* = 0.01559).

Even when excluding patients who passed away or experienced local disease progression before receiving radiotherapy, overall survival and local recurrence-free survival were reduced in patients who chose to forego radiotherapy (Figure 2 and Figure 3).

### 3.2. Chemotherapy

Neoadjuvant chemotherapy was recommended in 262 patients (32%). However, 40 (15%) individuals refused or did not undergo this recommended treatment. Within this group of patients, refusal was noted in 17 (6%) patients, while 20 (8%) demonstrated either disease progression, impaired wound healing, or medical reasons as cardiac decompensation. A total of 3 (1%) patients needed surgery preferred for local reasons as neurologic impairment.

Adjuvant chemotherapy was recommended in 285 (34%) patients. A total of 231 (81%) of those to whom it was recommended adhered to the recommended regimen. In total, 31 patients (11%) rejected adjuvant chemotherapy. Other impediments to the application of adjuvant chemotherapy included death of the patient (n = 4), wound healing problems (n = 6), deterioration (especially cardiac) in general health status (n = 4), or various other reasons.

In total, chemotherapy was advised in 334 (40%) patients, with 250 (75%) successfully completing all recommended cycles. A total of 25 (7%) individuals only received part of the recommended regimen, and 59 (18%) did not receive the recommended chemotherapy due to various reasons. In bone sarcomas, the refusal rate of CTx was 12.0% compared to 22.1% in soft tissue sarcomas (*p* = 0.0083). The mean age of patients who eventually received the recommended chemotherapy was 46 years, whereas those who refused had a mean age of 66 years (*p* < 0.001).

**Figure 4:**
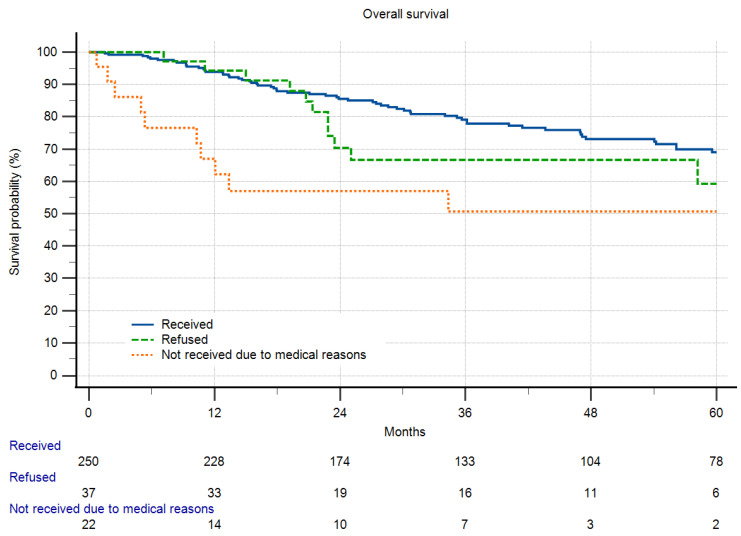
Overall survival in respect to chemotherapy and refusal of chemotherapy (*p* < 0.001). There was no significant difference between patients who refused and received CTx. Patients who did not receive CTx due to medical reasons did significantly worse than those who received CTx (*p* = 0.0009).

**Figure 5:**
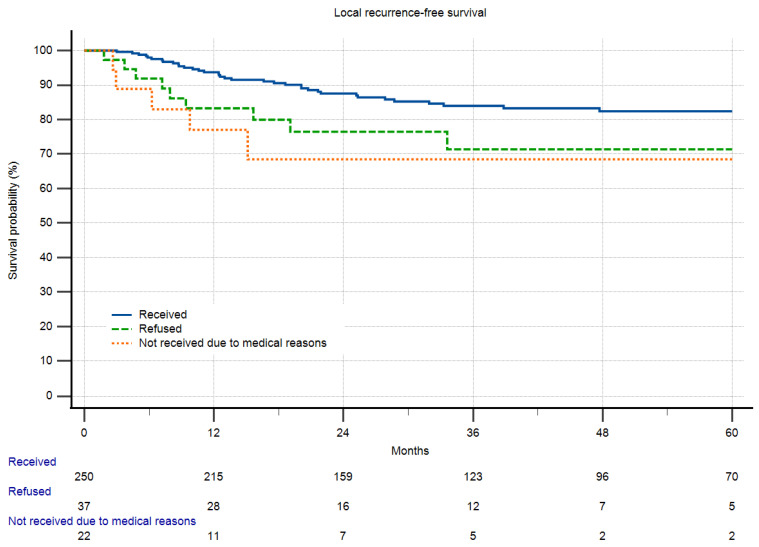
Local recurrence-free survival in relation to chemotherapy and refusal of chemotherapy (*p* = 0.0199). Both groups of patients refusing and not receiving CTx due to medical reasons did significantly worse than those who received CTx.

Local recurrence-free survival was significantly reduced in patients refusing chemotherapy. This effect was even more pronounced in patients who did not receive chemotherapy for legitimate medical reasons. Regarding overall survival, there was no significant difference between patients who refused and received CTx. Only those who did not receive CTx due to medical reasons did worse than other patients (Figure 4 and Figure 5). Refusal of therapy was independent of the grade of the disease for both radio- and chemotherapy.

In univariate analysis grading, primary/recurrent tumor, deep location, size > 5 cm, age, metastatic disease at surgery, and the refusal of radiotherapy proved to be significant factors for overall survival. In a multivariate analysis of the same factors, deep location and primary/recurrent tumor lost their significance (Table 3).

## 4. Discussion

Several studies have delved into patients with cancer, focusing on factors associated with their decision to refuse therapy [15]. However, in the realm of sarcoma therapy, there is a notable absence of thorough investigations addressing this particular aspect. Main reasons for refusal have been found to encompass advanced disease, poor performance status, and socio-demographic parameters such as gender, race, or insurance coverage [15,16]. Additionally, factors such as convenience, fear of side effects, and challenges related to transportation have been articulated [17,18]. In our study, insurance status could be excluded as a determining factor as all treatment modalities were financially covered by the German social insurance system. This is an important factor, as it has been shown in other countries that this might even influence therapeutic recommendations [19]. Also black race is shown to be associated with an increased refusal in cancer therapy [20]. In Germany, with an overwhelmingly white population, this is a negligible factor.

Age was significantly higher in individuals who refused therapy (CTx and RTx) in concordance with the existing literature. Only one comprehensive British study involving 1500 adults, delving into their prospective decision-making regarding cancer management, excluded age as a determining factor for therapy refusal [21]. We think that age is a surrogate factor for many other reasons such as general health status or inconvenience of traveling to providers of therapy, but we could not prove that in this study. In a 2011 Time publication, the near-certainty of side effects with only a possibility of a therapeutic effect was also considered as one of the relevant factors in refusing treatment [22]. This seems to be understandable from a common-sense point of view, especially in older patients with a higher risk of significant side effects. Jorgensen et al. were able to prove that in a study of patients refusing adjuvant chemotherapy for colorectal cancer. Quality of life and health status were considered more important in decision-making in older patients than in younger ones [23].

But one should also not forget that, besides refusal of the patient, the recommendation for adjuvant therapies such as radiotherapy itself might be also dependent on age of the patients. There is a high risk that older patients do not receive the same recommended treatment as younger ones. This was also significantly observed within our group of patients. In a study from Venigalla et al. of 6.978 patients with soft tissue sarcomas, 51% underwent surgery alone and 49% received pre- and postoperative irradiation. Elderly patients received radiation therapy less commonly than did non-elderly patients (49% vs. 52%) despite having higher-grade tumors and a higher frequency of positive resection margins [24].

Colson-Fearon et al. showed in a recent publication on risk factors and survival impact associated with refusal of radiotherapy in more than 210,000 patients with gynecologic cancers out of the National Cancer Database (NCDB), that refusal was positively associated with older age, native Hawaiian Pacific Islander race, and increased morbidity [25]. Less refusal was seen with Hispanic ethnicity, patients with insurance and an annual income of more than USD 74,000. RTx refusal was associated with a significantly lower survival. This seems to be a geographically independent observation also seen, for example, in China. Liu et al. published in 2023 that refusal of RTx in breast cancer patients was associated with age; socio-economic factors such as low income and divorce; white race (a minority); and advanced stage of the disease [26].

We expected that refusal of radiotherapy does influence local recurrence-free survival. Figure 3 shows, with the limitations of univariate Kaplan-Meier-analysis, such an effect. To our own surprise, refusing radiotherapy also was an independent factor for overall survival. Those with a recommendation for RTx clearly had tumors with higher risks. Patients receiving RTx came to the same outcome as those with no recommendation for RTx, but those who denied did worse. So, the question is, is radiotherapy an independent factor for overall survival in sarcoma patients? We cannot answer that with this study, but there is literature pointing in that direction [27].

In children, parents are most often the decisions makers. If their refusal might have a significant risk of harm to the child, then legal courts must decide [28]. This was not necessary in any of our cases but it may create a bias toward a higher age of refusing patients. As shown in a study by Caruso Brown et al. in 2017, this situation is complicated and not very well investigated [29]. Going to court means, in every case, a delay in therapy. A total of 51 of their collected cases (70%) involved some form of legal action. But legal action itself did not reliably predict survival. If possible, it should thus be avoided by using other methods [30].

Even marital status might influence the refusal of indicated therapies. Providing encouragement and much more, practical transportation is found to be a significant factor in this regard [31].

If patients refuse recommended therapies, one might assume that this would adversely impact their prognosis. In a heterogenous cohort of cancer patients within the SEER database, 2.2% out of more than 300,000 patients chose to decline radiotherapy. This decision correlated with a notable decline in overall survival, reducing the median survival time from 171 to 96 months [32]. However, it remains unclear whether factors such as performance status or disease progression influenced this outcome. Also, in this study, refusing radiotherapy was an independent factor for reduced overall survival.

Also utilizing also the SEER database, analogous results were substantiated in a population comprising 925,127 cancer patients. Among them, 0.4% refused surgery and 0.9% declined radiotherapy, which corresponded to an increase in cancer-specific mortality, with hazard ratios of 2.80 and 1.97, respectively [8]. This trend was corroborated in patients with head and neck cancer, where the refusal of postoperative radiotherapy resulted in comparable detrimental effects to those within our specific group of sarcoma patients [33].

In consideration of our own results, it is imperative to acknowledge that the compromised outcome observed in patients who did not receive radio- or chemotherapy due to medical reasons may stem from the cofounding influence of highly progressive disease or exacerbated general health conditions within this cohort. Specifically addressing chemotherapy, discernable effects on overall survival resulting from refusal of therapy without medical reasons were not evident. We think that this is because CTx has been shown to influence overall survival only in a group of selected high-risk patients, and then only in large prospective randomized studies [11]. This study is neither designed to measure that effect nor sufficiently powered to do so. Surprisingly, this did manifest a discernible effect on local recurrence-free survival, showcasing an approximate 10% difference. However, one may assume that the inclination toward expeditious surgical interventions was influenced by the presence of aggressive tumors that have a high potential for local and systemic recurrence. This scenario may have skewed the composition of the “compliant” group, featuring patients with a higher proportion of less aggressive lesions.

Refusing chemotherapy for non-medical reasons was observed to be nearly twice as prevalent (8.8%) compared to the refusal of radiotherapy (4.7%). This discrepancy might be attributed to the much greater complexity and demands associated with administering chemotherapy as opposed to radiotherapy. In addition, the time duration for radiotherapy is much shorter as for chemotherapy. In our setting, chemotherapy was recommended in conjunction with local hyperthermia. This forced the patients to undergo every cycle of chemotherapy as inpatient in a specialized center providing hyperthermia. Traveling distances to this center were sometimes great. Radiotherapy was applied in an outpatient fashion, in general in an institution close to the home of the patients. This higher proportion of denying CTx vs. RTx has also been shown in other cancers such as breast cancer. Of 2,058,568 cases in the US, 14.1% refused chemotherapy and 5.5% refused radiation [34].

Interestingly treating patients in centers might be one method to reduce the number of refusals. This was shown in a study on esophageal carcinoma and in head and neck cancer in the US [35,36]. To a lesser degree than other factors, treatment at low-volume centers was a factor predictive of treatment refusal.

There are a number of patients using complementary medicine at some point of their therapy for curable cancers. In a 2018 published study, this was also correlated with the refusal of additional conventional cancer treatment and was associated with a higher risk of death [37]. But one has to be aware that this might be a surrogate parameter combining several other psychological factors for declining conventional therapy modalities.

It is essential to underscore that this study was not designed to ascertain the specific effects of both forms of adjuvant treatment modalities in sarcoma patients. The study group was too heterogeneous and the subgroups would be underpowered for such investigation. However, what remains unequivocal is that refusal of recommended therapies, regardless of the rationale, is associated with detrimental effects on either local recurrence-free survival, overall survival, or both. This includes all mentioned forms of bias.

In essence, refusal of chemo- or radiotherapy is a decision influenced by various factors. Within the refusal group, a predisposition towards an older patient demographic or patients with deteriorated overall health status is invariably observed. To mitigate this inherent bias, we meticulously documented the distinct causes underpinning each individual’s refusal of therapy.

Reducing the time on adjuvant therapy is one option to overcome refusal in a number of patients. Radiotherapy in soft tissue sarcoma might be so administered in a hypofractionated protocol without disadvantages for the patient [38]. Also, including regional hyperthermia in radiotherapy might be an effective option for reducing treatment time in the most common adjuvant or neoadjuvant forms of sarcoma therapy [39]. Mitigating these circumstances necessitates enhanced communication of the benefits and disadvantages of recommended therapies. Such improved communication strategies could effectively reduce the underutilization of adjuvant or neoadjuvant therapies among sarcoma patients. This might need a change in attitude for many physicians. As published in 1989 in Israel, physicians do not try to persuade every patient [40]. The position of the physician depended not only on the diagnosis but also on the patient’s status and age. So, cofounding factors play an important role in the refusal of therapy. Also, in a study in the American Veterans Health Administration regarding the underuse of recommended therapies for colorectal and lung cancer, the physicians’ assessment of poor health of the patients was the major factor of not performing indicated therapies [41]. This was also seen as a bias in respect to age-related underuse.

Communication with patients is in many cases based on the wrong assumption of “informed refusal” or “informed consent”. Quite a number of the refusing patients do not weigh the pros and cons of the advocated therapies in the same rational way as the physicians [42]. Their personal values and perspectives are different, which makes it impossible, or at least very difficult, to discuss a comparative evaluation.

The literature shows that patients refusing advocated therapies have a wrong assumption of the pros of the advised treatment options and, in some cases, a general mistrust in the healthcare system [43]. To overcome this and to enhance communication with patients, an institutional psycho-oncologic team has been integrated in the general setting of sarcoma (and also cancer) therapy in our center.

In summary, communication with cancer patients has been proven to affect decision-making, patient satisfaction, patient distress, patient compliance, and also malpractice litigation [44].

We have identified prognostic factors that are independently associated with a significant deterioration in prognosis in the setting of therapy refusal. More specifically, we have identified a group of patients for whom refusal of treatment would therefore be particularly bad. To receive or deny therapy is the only factor that patients themselves can influence in the setting they are in. These are individuals with high-grade tumors, tumor size > 5 cm, older age, and metastatic disease at surgery. In those patients, the utmost effort should be made to convince them to stick to the established therapeutic recommendations.

## 5. Limitations of This Study

This study is retrospective. The patients were originally enrolled prospectively but the reasons for therapy refusal were either collected from medical records or through retrospective interviews with patients or their relatives. Due to this, it was not possible to do a more profound interview in those patients refusing further therapies. Our study only included sarcoma patients but it included those with all sarcoma entities. We only had the chance to evaluate overall survival, as data on disease-specific survival could not be obtained. Hence, age as a predictive factor on prognosis has to be seen with this limitation.

Although we think that this study’s inclusion criteria are suitable for evaluating the proportions of therapy refusals, it is acknowledged that it might influence the outcome. Regarding other possible factors as ethnicity, religion, and level of education, or the possibility of homing in a more rural or city area, we were unable to collect these data for several reasons, but we have provided the relevant information obtained by other authors in the discussion section.

## 6. Conclusions

Refusing chemotherapy for non-medical reasons was seen in 8.8% of study patients, and refusal of radiotherapy in 4.7%. In total, 10% of the patients did not receive the recommended radiotherapy, and 25% received either none or less than the recommended chemotherapy. This deteriorated overall survival as well as local recurrence-free survival across both therapeutic modalities. To mitigate this effect, enhanced communication with patients is imperative for reducing the underutilization of adjuvant or neoadjuvant therapies in sarcoma patients. Reducing treatment times, as achieved with hypofractionated radiotherapy and therapy in a high-volume sarcoma center, might also have a positive effect on patient compliance with the treatment recommendations.

## Figures and Tables

**Table 1:** Distribution of histiotypes.

Entities	N	%
Undifferentiated pleomorphic sarcoma (UPS)	164	20%
Liposarcoma	152	18%
Chondrosarcoma	113	14%
Osteosarcoma	86	10%
Myxofibrosarcoma	68	8%
Leiomyosarcoma	54	7%
Synovial sarcoma	36	4%
Ewing sarcoma	26	3%
Malignant peripheral nerve sheath tumor (MPNST)	23	3%
Fibrosarcoma	21	3%
Other lesions	85	10%

**Table 2:** Tumor characteristics at time of definitive surgery of the primary or the recurrent disease.

Tumor Characteristics	N	%
Soft tissue	609	74%
Bone	219	26%
Primary disease	691	83%
Recurrent disease	137	17%
Deep	735	89%
Superficial	93	11%
Size > 5 cm	491	59%
Size ≤ 5 cm	337	41%
G1 *	187	23%
G2 *	249	30%
G3 *	345	42%
GX ^+^	47	6%
No metastatic disease	756	91%
Metastatic disease	72	9%

* According to the Fédération Nationale des Centers de Lutte Contre le Cancer (FNCLCC) system [10]; ^+^ Grading not applicable.

**Table 3:** Multivariate Cox proportional-hazards regression of factors influencing overall survival.

Factor	*p*	HR	95% CI
Grading	<0.0001	2.16	1.70–2.73
Primary/Recurrent tumor	0.0513	1.43	1.00–2.05
Deep location	0.3814	1.30	0.72–2.34
Size > 5 cm	0.0001	2.11	1.47–3.04
Age	<0.0001	1.03	1.02–1.04
Metastatic disease at surgery	<0.0001	4.05	2.72–6.01
Refusal of RTx	<0.0001	3.05	1.93–4.82
Refusal of CTx	0.1515	0.64	0.34–1.18

HR—hazards ratio; CI—95% confidence interval.

## Data Availability

The datasets used and/or analyzed during the current study are available from the corresponding author on reasonable request.

## Abbreviations

CTx: chemotherapy; EURAMOS: European and American Osteosarcoma Study Group; EURO-B.O.S.S.: EUROpean Bone Over 40 Sarcoma Study; EURO-EWING: EUROpean Ewing tumour Working Initiative of National Groups; G1, G2, G3: Grading according to the Fédération Nationale des Centers de Lutte Contre le Cancer (FNCLCC) system; R0, R1, R2: resection margin; RTx: radiotherapy; SD: standard deviation; SEER: Surveillance, Epidemiology, and End Results.
